# Carcinoembryonic Antigen Related Cell Adhesion Molecule 6 Promotes Carcinogenesis of Gastric Cancer and Anti-CEACAM6 Fluorescent Probe Can Diagnose the Precancerous Lesions

**DOI:** 10.3389/fonc.2021.643669

**Published:** 2021-06-17

**Authors:** Fangmei An, Chuwei Zheng, Guoqiang Zhang, Liangyun Zhou, Yuqing Wu, Zheng Hou, Zhiyi Zhou, Ke Chen, Qiang Zhan

**Affiliations:** ^1^ Department of Gastroenterology, Wuxi People’s Hospital Affiliated to Nanjing Medical University, Wuxi, China; ^2^ Suzhou Institute of Biomedical Engineering and Technology, Chinese Academy of Sciences, Suzhou, China; ^3^ Department of Pathology, Wuxi People’s Hospital Affiliated to Nanjing Medical University, Wuxi, China

**Keywords:** CEACAM6, fluorescent-labeled probe, near infrared-labeled probe, gastric cancer, precancerous lesions

## Abstract

The diagnosis of precancerous lesions or early gastric cancer (EGC) is very important for patient survival. Molecular imaging is a visualized method that can easily and precisely diagnose tumors. However, there are currently few studies about molecular imaging diagnosis of EGC. Here, we studied the expression of carcinoembryonic antigen related cell adhesion molecule 6 (CEACAM6) in the progression of GC. Then, the regulatory roles of CEACAM6 in GC cells were investigated. Furthermore, both the fluorescent-labeled and near infrared molecular-labeled probes were synthesized, and the diagnostic value of anti-CEACAM6 probes in GC was evaluated *in vivo* using a GC mice model as well as *in vitro* using fresh dysplastic gastric mucosa obtained from endoscopic submucosal dissection (ESD) operations. Our study showed that CEACAM6 was over expressed in GC tissues compared to adjacent tissues, and the patients with higher CEACAM6 expression had lower survival time. Moreover, the CEACAM6 expression was higher in the dysplastic gastric mucosa than in the adjacent normal mucosa. CEACAM6 accelerated the growth, proliferation, and invasion of GC cells in the *in vitro* and *in vivo* studies. Moreover, up regulated CEACAM6 can induce the expression of proteins related to GC progression. Furthermore, the anti-CEACAM6 probes exhibited good affinity with GC cell lines. The probes can track tumors as well as metastases in the mice model *in vivo*, and can precisely identify the area of dysplastic gastric mucosa using specimens obtained from ESD operations by wide field fluorescent endoscopy. The surface micro features of the mucosa can also be observed using fluorescent micro endoscopy, and the degree of atypia can be distinguished by both the signal intensity and surface micro morphology. CEACAM6 is a key molecular marker in GC progression, and the anti-CEACAM6 probe-assisted fluorescent endoscopy may be a potential option for the diagnosis of precancerous lesions.

## Introduction

Gastric cancer (GC) is one of the leading causes of cancer-related deaths worldwide (1). The morbidity and mortality of GC in China have long been at the forefront globally (2, 3). Early GC (EGC) has a 5-year survival rate that is more than 90%; by contrast, it is less than 20% in patients with advanced GC (AGC) (4, 5). Therefore, it is important to diagnose GC at the very early stages. Endoscopy-assisted pathology is currently the key diagnostic method for EGC; however, the preneoplastic lesion features are sometimes atypical under endoscopy in some cases. Although the new type of endoscopies such as high-resolution endoscopy, chromoendoscopy, magnification endoscopy, fluorescence endoscopy, narrow-band imaging, optical coherence tomography, point spectroscopy, and confocal laser endomicroscopy have been developed very well, the positive detection rate often depends on the experiences of operators. Therefore, it may sometimes be difficult to find the lesions. Hence, finding an easy and precise method to detect lesions during EGC is of urgent importance.

The emergence of “molecular imaging” have led to a boom in studies regarding tumor diagnosis (6, 7), the specificity of antibodies for antigens over expressed or uniquely expressed in tumor cells makes them ideal candidates in the development of bioconjugates for tumor imaging, the molecular probes are being used to detect tumors or neoplastic lesions through fluorescent endoscopy and are being widely applied in surgical navigation (8). The studies had investigated that the carcinoembryonic antigen (CEA) targeted fluorescent imaging was successfully used in both pancreatic and colorectal cancer (9), In 2018, the carcinoembryonic antigen related cell adhesion molecule5 (SGM-101) fluorescent probe was used in the diagnosis of pancreatic cancer and the metastases, with an accuracy of 84% and sensitivity of 98% (10). Nevertheless, similar investigations in EGC diagnosis remain insufficient. CEACAM6 is one of the members of CEA family, and is a protein expressed in the cell membrane (11). It is overexpressed in the tumor tissues of GC and has been found to be closely related to the angiogenesis and metastasis of GC (12). In 2016, a study showed that the fluorescent-labeled anti-CEACAM6 probe (CEACAM6-Alexa Fluor488) can label the tumors in patient-derived tumor xenografts (PDTX) (13), however, its diagnostic value in preneoplastic lesion has not yet been investigated.

In the present study, the CEACAM6 expression in the progression of gastric mucosa carcinogenesis was checked, and the regulated role of CEACAM6 in GC was studied *in vitro* as well as *in vivo*. Furthermore, the diagnostic role of the anti-CEACAM6 probe was investigated in a GC mice model and in dysplastic gastric mucosa specimens from ESD operation.

## Materials and Methods

### Human Tissue Samples Collection

The gastric mucosa specimens were divided into four groups according to the pathology results (1): Chronic Superficial Gastritis (CSG) group, characterized by the gastric mucosa having lymphocytes and plasma cell infiltration without changes in atrophy or intestinal metaplasia (2); Chronic Atrophic Gastritis (CAG) + Intestinal Metaplasia (IM) group, characterized by atrophy of the intrinsic glands in the gastric mucosa and by gastric mucosal epithelium that has transformed into the mucosal epithelium of the small or large intestine containing goblet cells (3); Dysplasia (Dys) group, also is preneoplastic lesion, characterized by gastric mucosal epithelium and glands that have deviated from normal differentiation and were atypical in shape and function, and can be divided in two three subgroups depending on the degree of atypia (mild, moderate, and severe); and (4) Gastric Cancer (GC) group, characterized by AGC lesions with cancer cells invasion beyond the submucosa to the muscularis propria or beyond. Gastric mucosa specimens were collected from CSG, CAG+IM, Dys, and GC patients through biopsy during their endoscopy procedures at the Wuxi People’s Hospital between October and December 2019. Three samples were collected for each group, and total 12 samples were kept in RNAlater (AM7020, Thermo Fisher Scientific) for the mRNA high throughput sequencing (HTSeq v0.11.2) analysis and reverse transcription‑quantitative polymerase chain reaction (RT‑qPCR) verification.

A total of 15 fresh specimens (mild=5, moderate=5, severe dysplasia=5) obtained from the ESD procedures at the Wuxi People’s Hospital between January and December 2020 were used for the *in vitro* probe studies, and then the specimens were fixed with formalin for the HE and IHC staining.

A total of 89 tumor and matched adjacent tumor tissues (62 from male patients and 27 from female patients) were collected from GC patients during their surgical procedures at the Wuxi People’s Hospital between January 2013 and December 2014 (the clinical data are shown in **Table 1**). The tissue chips were made by Shanghai ZuoCheng Bio Co., Ltd (Shanghai, China). All patients had not received chemotherapy or radiotherapy before their surgeries. The UICC TNM method was used for pathological classification (14), and all of the tissue chips were used for IHC staining.

**Table 1:** Correlations of CEACAM6 mRNA expression with different clinic-pathological factors.

Subtypes		CEACAM6	
		HR (95%CI)	*P value*
Gender	male	0.95 (0.77 − 1.17)	0.6236
	female	0.94 (0.67 − 1.34)	0.7394
Clinical stage	1	0.58 (0.21 − 1.59)	0.2817
	2	0.42 (0.22 − 0.78)	**0.0049**
	3	0.95 (0.71 − 1.26)	0.7284
	4	1.15 (0.78 − 1.68)	0.4822
T stage	1	/	/
	2	0.7 (0.46 − 1.07)	0.0999
	3	1.08 (0.76 − 1.52)	0.6716
	4	0.65 (0.28 − 1.52)	0.3124
N stage	0	0.47 (0.2 − 1.11)	0.0771
	1	0.64 (0.42 − 0.98)	**0.0364**
	2	1.1 (0.7 − 1.72)	0.6888
	3	1.13 (0.67 − 1.93)	0.6424
M stage	0	0.8 (0.61 − 1.05)	0.1127
	1	1.32 (0.74 − 2.35)	0.3467
Lauren classification	intestinal	0.75 (0.55 − 1.03)	0.0747
	diffuse	0.84 (0.6 − 1.18)	0.3145
	mixed	1.1 (0.4 − 3.04)	0.8541
Differentiation	poorly	1.49 (1 − 2.22)	0.0505
	moderately	1.57 (0.82 − 3.03)	0.1736
	well	0.78 (0.33 − 1.84)	0.5664
HER2 status	negative	0.97 (0.78 − 1.21)	0.7962
	positive	0.7 (0.54 − 0.91)	**0.0079**
Treatment	surgery	0.89 (0.67 − 1.19)	0.438
	5-FU based adjuvant	1.44 (1.02 − 2.04)	0.0395
	Other adjuvant	0.47 (0.19 − 1.2)	0.1069

Bold values, P < 0.05.

### Cell Culture, Induction and Infection

The human GC lines AGS, MKN-45, and normal gastric epithelial cell lines GES-1 were purchased from the cell bank of the Chinese Academy of Sciences (Shanghai, China). Cells were cultured in Dulbecco’s modified Eagle medium (DMEM) supplemented with 10% fetal bovine serum (FBS), 100 U/mL penicillin, and 100 g/mL streptomycin separately, the cells were culture in a 37°C humidified incubator with 5% CO2.

Src inhibitor was purchased from MCE MedChenExpress (cat no.179248-59-0), it was dissolved with dimethyl sulfoxide (DMSO) and got the finial concentration of 10μM. The 2.5×10^5^/ml AGS or MKN-45 cells were seeded in 6-well plates, 2ml/well, after 24h, the 2μl solution was added into cells/well, the control group was added the DMSO only, after 24 h, the medium was changed and the induced cells were infected with lentivirus for the following studies.

CEACAM6 overexpression lentivirus (Lenti-CEACAM6) and CEACAM6 RNAi lentivirus (CEACAM6 RNAi) with fluorescein expression purchased from Genechem company (Shanghai Genechem Co., Ltd), the AGS, MKN-45, and GES-1 cells were seeded in 6-well plates to reach about 30% confluence. HiTransG A (60 µl) and 7×10^8^ TU/ml CEACAM6 lentivirus (Lenti-CEACAM6, CEACAM6 RNAi) or negative control lentivirus (Lenti-NC) (4 µl) were added to 1.5 ml of the complete medium. After 12 h of incubation, the medium was replaced with the complete medium for 72 h for the *in vitro* and *in vivo* experiments described below.

### Hand-Held Fluorescent Endoscopy and Micro Endoscopy Detectors

The hand-held detectors of wide field multispectral microscope and a high-resolution microendoscope were supplied by the Suzhou Institute of Biomedical Engineering and Technology, Chinese Academy of Sciences (Suzhou, China).

### Identification of CEACAM6 mRNA Expression Using TCGA Dataset

To investigate the GC CEACAM6 mRNA expression in published works, we performed a search in TCGA (http://ualcan.path.uab.edu) using the combined keywords “CEACAM6 AND stomach adenocarcinoma.” The CEACAM6 expression in different clinic stages of GC was also investigated.

### Total RNA Extraction, mRNA Sequencing Analysis and RT‑qPCR

Total RNA was extracted from the gastric mucosa specimens of patients using Trizol (Invitrogen, Grand Island, NY, USA), according to the manufacturer’s instructions. The QIAGEN QIAseq FastSelect RNA Removal Kit (QIAGEN, Germany) was used to remove rRNA and achieve RNA fragmentation (average fragment length of approximately 200 nt). The purified RNAs were subjected to first strand and second strand cDNA synthesis following by adaptor ligation and enrichment with a low-cycle according to instructions of NEBNext^®^ Ultra™ RNA Library Prep Kit for Illumina (NEB, USA). The purified products were evaluated using the Agilent 2200 TapeStation and Qubit^®^2.0(Life Technologies, USA) and then diluted to 10 pM for cluster generation *in situ* on the pair-end flow cell followed by sequencing (2×150 bp) HTSeq.

The clean reads were obtained after removal of reads containing adapter, ploy-N and at low quality from raw data. HTSeq was subsequently employed to convert aligned short reads into read counts for each gene model. Differential expression was assessed by DEGseq using read counts as input. The Benjamini–Hochberg multiple test correction method was enabled. Differentially expressed genes were chosen according to the criteria of fold change > 2 and adjusted q-value < 0.05. All the differentially expressed genes were used for volcano map analysis, the expression of CEACAM6 mRNA was verified by RT‑qPCR using the same total RNA for the sequencing above.

RT‑qPCR primer set for CEACAM6 (MQPS0000872‑1‑100) and β-actin (snRNa, MQPS0000002‑1‑100) purchased from Guangzhou RiboBio Co. Ltd. A total of 0.5 µg total RNA was used to synthesize cDNA in a 20 µl reaction volume at 42°C for 45 min using a PrimeScriptsis kit (cat. no. RR047a; Takara Bio, Inc.). RT‑qPCR was performed in a 20 µl reaction volume using iQ™ SYBR Green Supermix (cat. no. 1708882aP; Bio‑Rad Laboratories, Inc.) The thermocycling conditions were as follows: 95°C for 3 min, followed by 40 cycles of 95°C for 20 sec, 60°C for 30 sec, and 70°C for 30 sec. The β-actin was used as the internal control for CEACAM6, which was quantified using the 2^‑ΔΔCt^ method. All RT‑qPCR experiments were performed in triplicate.

### IHC Staining

The tissues were fixed and dehydrated, followed by paraffin embedding and cutting. The 5 µm-thick sections were rehydrated and subjected to antigen retrieval buffer, followed by incubation with primary antibodies against CEACAM6 (1:40, cat. no., ab275022, Abcam, USA), p-Akt (1:100, cat. no. 4060, CST, USA), p-PI3K (1:200, cat. no. 17366, Abcam, USA), p-Src (1:50, cat.no. 2105S, CST, USA), and MMP9 (1:100, cat.no. ab58803, Abcam, USA) for 1 h at room temperature. After washing thrice with tris-buffered saline (TBS), the sections were incubated with Envision-labeled polymer-horseradish peroxidase (HRP)-conjugated rabbit antibody (1:2,000; cat. no. 7074; Cell Signaling Technology, Inc.) for 1 h at room temperature, and were then visualized using diaminobenzidine.

All immunohistochemical sections were scanned by 3D HISTECH (pannoramic MIDI) and 10 randomly selected fields were checked under pannoramic viewer, the percentage of positive cells was analyzed by the densito quant software. The staining intensity was: strongly positive showed in dark brown was 3, the moderately positive showed in brown was 2, the weakly positive showed in light yellow was 1 and the negative showed in blue was 0. The positive cell percentage was calculated, and finally the histochemistry score (H-Score) was calculated. H-Score = ∑ (PI ×I) = (percentage of cells of weak intensity ×1) + (percentage of cells of moderate intensity ×2) + percentage of cells of strong intensity ×3), where PI is the percentage of positive cells in all cells in the section, I is the staining intensity.

All histopathological sections were read, diagnosed, and recorded by two senior pathologists.

### Cell Invasion

For the cell invasion assay, 2.5×10^4^ infected AGS and MKN-45 cells obtained as mentioned above were seeded in 24-well plates with inserts. The two-chamber system was equipped with a cell-permeable membrane coated with Matrigel (R&D Systems, USA). After culturing for 48 h, cells that invaded through the matrigel and reached the bottom of the insert were fixed with formalin, and stained with crystal violet, the cells in the bottom chamber were considered invaded and were counted using Image-Pro Plus software.

### Cell Proliferation

AGS and MKN-45 cells infected with Lenti-CEACAM6 or Lenti-NC were seeded in 96-well plates with 10^4^ cells/well, with three replicates in each group. After 48 h in complete medium, cell proliferation was detected using Lights’ EdU Apollo567 kit (cat. no.C10310-1; Guangzhou RiboBio Co. Ltd) according to the manufacturer’s instructions. The cells were observed under fluorescence microscope, with the total live cells in blue and the proliferated cells in red. The cells number were estimated using Image-Pro Plus software and the proliferation rate= (number of proliferated cells/total number of cells) ×100%.

### Protein Extraction and Western-Blot

Cells were harvested and then lysed with radioimmunoprecipitation assay buffer (Pierce; Thermo Fisher Scientific, Inc.). The protein concentration was determined using the bicinchoninic acid assay, and proteins were separated using 10% SDS‑PAGE and were transferred onto polyvinylidene difluoride membranes. The membranes were blocked with 5% skimmed milk in TBS‑Tween‑20 (TBST), followed by overnight incubation at 4°C with rabbit anti-human CEACAM6 (1:500, cat. no. ab275022, Abcam, USA), Akt (1:1000, cat. no. 9272, CST, USA), p-Akt (1:2000, cat. no. 4060, CST, USA), PI3K (1:1000, cat. no. 4255, CST, USA), p-PI3K (1:500, cat. no. 17366, Abcam, USA), MMP9 (1:500, cat. no. ab58803, Abcam, USA), and p-Src (1:1000, cat.no.2105S, CST, USA), Src (1:1000, cat.no. 2109T, CST, USA), β-Tubulin (1:1000, cat. no.2146S, CST, USA) and β-actin (1:1000, cat. no.4970S, CST, USA) antibodies at 4°C. The membranes were washed with TBST thrice, followed by incubation with HRP-conjugated goat anti-rabbit IgG (cat. no.7074, Sigma-Aldrich) for 1 h at room temperature. The protein bands on the membrane were visualized using the SuperSignal West Pico Chemiluminescent Substrate (Pierce) and were quantified using Image-Pro Plus software.

### Anti-CEACAM6 Antibody Synthesis

The CEACAM6 monoclonal antibody (CEACAM6 mAb; NP_002474.4; Met 1-Gly 320) was purchased from Sino Biological (cat. no.10823-R408). CEACAM6 mAb was obtained from a rabbit immunized, the gene of antibody was characterized by phage display technology, and then, the targeted gene was constructed into eukaryotic expression vector, the vector was transfected into the 293 cells, lastly, the antibody was expressed and purified.

### CEACAM6-mAb-Alexa Fluo488 Probe Synthesis

0.1 mg Alexa Fluo 488 was dissolved into 0.1 ml PBS(pH=8.4)buffer solution in the dark room, then 1 ml anti-CEACAM 6 mAb (1 mg/ml, cat. no.10823-R408, Sino Biological) was added into the above solution, mixed together and placed at room temperature for 2 h, then the mixture was centrifuged thrice at 1200 rpm using a desalination column (Pierce Zebra, 1 ml) and 0.01 M PBS was used as the mobile phase. The filtrate was collected and the anti-CEACAM6-mAb-Alexa Fluo488 probe was obtained.

### CEACAM6-mAb-IR Dye 800CW NHS Probe Synthesis

0.5 mg IR Dye 800CW NHS was dissolved into 0.5 ml PBS (pH=8.4) buffer solution in the dark room, then 5 ml anti-CEACAM 6 mAb (1 mg/ml, cat. no.10823-R408, Sino Biological) was added into the above solution, mixed together and placed at room temperature for 2 h, then the reaction, the mixture was centrifuged thrice at 1200 rpm using a desalination column (Pierce Zebra, 5 ml) and 0.01M PBS was used as the mobile phase. The filtrate was collected and the CEACAM6-mAb-IRDye 800CW probe was obtained.

### CEACAM6-mAb-Alexa Fluo488 Probe Affinity Testing in Cells

AGS, MKN-45, and GES-1 cells were cultured in dishes for 24 h for the confocal experiment. Then, the cells were fixed with 4% paraformaldehyde at 4 for 20 min, blocked with goat serum for 1 h at room temperature, and incubated with CEACAM6- mAb-Alexa Fluo488 probe synthesized as described above (1:20) at 4°C overnight. Finally, the nuclei were stained with DAPI and the cells were observed under confocal microscope. The average fluorescence intensity was calculated using the Image-Pro Plus software (Media Cybernetics, Rockville, MD, USA).

### CEACAM6 mAb-IRDye800CW Activity Testing by ELISA Method

In order to test if the conjugate (IRDye800CW NHS) affect the activity of the antibody, the ELISA (Enzyme Linked Immunosorbent Assay) method was applied. The recombinant CEACAM6 protein (CD66c, cat. no. 10823-H08H, Sino Biological) was dissolved with PBS, then put the 50ul CEACAM6 dissolving solution (100ug/ml) into the 4950ul coating buffer and got the coating solution (1ug/ml). Put the coating solution into the 96-well ELISA plates (100ul/well) and incubated at 4°C for 20 h, then the plates were washed for three times and blocked with 1%BSA-PBST blocking buffer at 37°C for 2 h, after washing, the CEACAM6 mAb or CEACAM6 mAb-IRDye800CW with different dilution times was added into the 96-wells (100ul/well), and incubated at 37°C, after 2 h, the plates were washed and the anti-rabbit secondary antibody was added into the wells (100ul/well), after incubating at room temperature for 1 h, the plates were washed, the color was developed with tetramethylbenzidine (TMB) ELISA substrate (100ul/well) (abcam,ab171523) and terminated with 1M H_2_SO_4_ (50ul/well), finally the OD value was read at 450nm.

when calculating half maximal inhibitory concentration (IC50) value, subtracted the OD value when the concentration of antibody is 0 from each OD value, then the value obtained by subtraction was input into graphpad prism 8.0.2 software for the curve fitting, and the IC50 value was got according to the fitting curve, the lower the IC50, the higher the activity of the antibody.

### CEACAM6 mAb-IRDye800CW Affinity Testing by Surface Plasmon Resonance (SPR) Analysis

#### Immobilization of Human CEACAM6 (CD66c) Protein

The coupling was did according to the manufactory’s instruction (cytiva, cat no. BR100050). The HBS-EP(10X, containing 0.1 M HEPES, 1.5 M NaCl, 30 mM EDTA and 0.5% v/v Surfactant P20)was used as running buffer, the flow cells Fc1 and Fc2 of Series S Sensor Chip (cytiva, cat. no. 29149603) were activated with a fresh mixture of 11.5 mg/ml NHS and 75 mg/ml EDC (1:1) for 420s, 5μg/ml of human CEACAM6 protein (CD66c, cat. no. 10823-H08H, Sino Biological) was diluted with 10 mM sodium acetate pH 4.0 (Cytiva, cat no. BR100349), and then injected to Fc2 at 10μl/min for 420s. The remaining active coupling sites of Fc1 and Fc2 were blocked with injection of 1 M Ethanolamine hydrochloride-NaOH for 420s, Fc1 was a reference channel in subsequent experiments. The final immobilization level of Fc2 reached to about 34.4RU.

### Kinetics Measurement Between CEACAM6 (CD66c) and CEACAM6 mAb or CEACAM6-mAb-IRDye800CW Antibody

The kinetic measurement was performed at 25°C. HBS-EP buffer (10X) was used to dilute CEACAM6 mAb or CEACAM6-mAb-IRDye800CW antibody, 20μg/ml was the highest concentration, 2-fold dilution of 20μg/ml till got 0.3125μg/ml. The gradient diluted antibodies were injected to Fc1 or Fc2, the flow rate was 30µl/min, the association time was 180s and dissociation time was 600s. 10mM glycine HCl (pH 1.5, cytiva, cat no.BR100354) was selected to regenerate the combined CEACAM6 mAb or CEACAM6-mAb-IRDye800CW antibody, and the regeneration time was 30s. Different concentrations of the antibody were injected repeatedly to bind, dissociate and regenerate. Finally, the association (Ka) and dissociation (Kd) rate constants were evaluated using Biacore T200 evaluation software (GE Healthcare Bio-sciences, Sweden), and the average dissociation constant (KD= Kd/Ka) was got. The 1:1 binding model was used for evaluating the binding of CEACAM6 mAb or CEACAM6-mAb-IRDye800CW to CEACAM6 (CD66).

### Subcutaneous Tumor Xenograft Model

Female nude mice (6–8 weeks old) were provided by the Changzhou Kaiwen Laboratory Animal Co., Ltd. (Jiangsu, China). The mice were maintained under specific pathogen-free conditions and were housed in ventilated cages with free access to food and water. The mice were allowed to acclimate for one week. They were anesthetized with 2% isoflurane for euthanasia and the cervical dislocation method was used to confirm death. The animal procedures were carried out in accordance with the guidelines of the Nanjing Medical University Laboratory Animal Center.

MKN-45 cells transfected with Lenti-CEACAM6, CEACAM6-RNAi or Lenti-NC were cultured in 10% FBS-containing DMEM and were then harvested. Approximately 4×10^7^ cells were resuspended in 100 µl of saline and were subcutaneously inoculated into the mice. 30 days after inoculation, when the tumors have grown to about 200 mm^3^, the tumor volume (0.5×length×width^2^) was recorded every 5 days, and the mice were used for the *in vivo* imaging study described below, the tumor and liver tissues were harvested to HE and IHC staining.

### *In Vivo* Mice Imaging

The CEACAM6mAb-IRDye800CW was injected through the tail vein using the following doses: 2.5 mg/kg, 5 mg/kg, and 10 mg/kg. The animals were observed at 2 h, 1 d, 2 d, 4 d, and 7 d after the injection, and were anesthetized with sodium pentobarbital by intraperitoneal injection at a dose of 40 mg/kg. After several minutes, the animals were placed in the *in vivo* imaging system (IVIS) (PerkinElmer) for near infrared imaging. The animals were euthanized after the last examination, with the cervical dislocation method used to confirm death, then the tumor as well as other organs were removed for near infrared imaging. The data were collected and analyzed using living image software (Xenogen) according to the manufacturer’s instructions.

### Diagnosis Value of the CEACAM6 mAb-IRDye800CW Probe to Specimens Obtained From ESD

The CEACAM6 mAb-IRDye800CW probe was sprayed on the surface of fresh tissues obtained from the ESD procedure, staining for 30 min at room temperature. Then, the tissues were washed thrice with running water and the fluorescent probe was completely removed from the surface. Fluorescence was checked using the fluorescent endoscopy detector and the surface microstructure was observed using detector of the fluorescence microscopic endoscopy detector.

### Statistical Analyses

All experiments were performed in triplicate, and all data were expressed as mean ± standard error. GraphPad Prism 5 software (GraphPad Software, Inc., GPW5-384305-RAG-5235) was used to perform statistical analyses. Differences between two groups were analyzed using the unpaired t-test, and the one-way analysis of variance (ANOVA) followed by Bonferroni’s *post hoc* test was used to analyze the differences between more than two groups. The paired datasets (adjacent tumor and tumor) were analyzed using the Wilcoxon matched-pairs signed rank test. A p value <0.05 was considered statistically significant. The correlation between fluorescence intensity and CEACAM6 staining H-Score was studied by calculation of Pearson correlation coefficients. Kaplan-Meier survival curve analysis was used to identify the difference between patients with high and low CEACAM6 expression, and the Cox risk proportional regression model was used to determine the GC risk factors.

## Results

### The Expression of CEACAM6 mRNA in Gastric Mucosa From Surface Gastritis to Cancer

**Figure 1:**
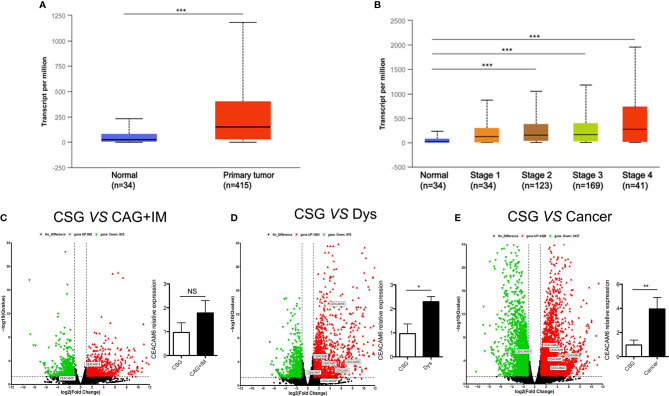
CEACAM6 mRNA expression in the gastric mucosa from normal to cancer. **(A)** Searched the CEACAM6 mRNA expression in the normal and stomach adenocarcinoma tissues in TCGA dataset through UALCAN (http://ualcan.path.uab.edu) website, normal (n=34), primary tumor (n=415). **(B)** Searched the CEACAM6 mRNA expression in the stomach adenocarcinoma tissues with different clinic stage in TCGA dataset, normal (n=34), stage 1 (n=34), stage 2 (n=123), stage 3 (n=169), stage 4 (n=41). The volcano map showed the deregulated mRNAs in CAG+IM **(C)**, Dys **(D)** and Cancer **(E)**, the red triangles represented up regulated mRNAs and the green triangles represented down regulated mRNAs (P<0.05), the deregulated mRNAs had no significant differences were showed in the black triangles, the up or down regulated CEA family members were verified by RT-qPCR and labeled in the map. The histograms in the volcano map showed the verification of CEACAM6 mRNA expression. *P < 0.05, **P < 0.01, ***P < 0.001, NS, no significance. CSG, Chronic Superficial Gastritis; CAG+IM, Chronic Atrophic Gastritis (CAG) + Intestinal Metaplasia (IM); Dys, Dysplasia; Cancer, Gastric Cancer.

Data from TCGA showed that CEACAM6 mRNA is up regulated in cancer tissues compared to their levels in normal tissues in stomach adenocarcinoma (**Figure 1A**). Furthermore, the CEACAM6 expression in cancer tissues with clinical grades 2, 3, and 4 are much higher than in the normal tissues (**Figure 1B**). However, there are currently few studies about CEACAM6 expression during GC progression. Thus, in order to investigate the expression and possible role of CEACAM6 in gastric mucosa carcinogenesis, the mRNA sequencing was performed on gastric mucosa specimens with CSG, CAG+IM, Dys, and Cancer, and the genes of CEA family member were verified by RT-qPCR method (only the data of CEACAM6 mRNA was showed). Compared with CSG, there were 882 up regulated and 633 down regulated mRNAs in the CAG+IM group, and among these mRNAs, the CEACAM18 was verified up regulated and CEACAM21 was down regulated (**Figure 1C**). Moreover, there were 1581 up regulated and 876 down regulated mRNAs in the Dys group, and among these, CEACAM1, 3, 4, 6, 18, 20, and 22 were verified up regulated (**Figure 1D**). In the Cancer group, 4429 mRNAs were up regulated and 2937 mRNAs were down regulated, and among these, CEACAM 3, 4, 5, 6 and 20 were verified up regulated and CEACAM19 was down regulated (**Figure 1E**). From the data above, it was showed CEACAM6 was up regulated (P<0.05) in both Dys and Cancer groups. The mRNA sequencing data has been submitted to the GEO repository and the series record is GSE163416.

### CEACAM6 Protein Expression in the Gastric Mucosa Among Patients With Cancer or Dysplasia

**Figure 2:**
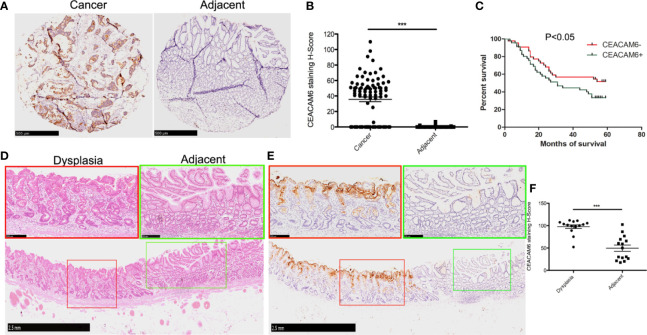
The CEACAM6 expression in the cancer tissues and dysplastic gastric mucosa. **(A)** Immunohistochemical staining (IHC) was performed to examine the expression of CEACAM6 in tumor and tumor-adjacent tissues using tissue chips. The tissues expressed CEACAM6 were stained in brown. Scale bars = 500 μm. **(B)** The histograms showed the quantification of IHC staining. Bar represents the Mean (± SEM) of staining H-Score (n=89). ***P<0.001. **(C)** The survival curve was drawn according to the CEACAM6 expression data got from the IHC analysis above. **(D)** HE staining was performed on the ESD specimen, the normal mucosa had the regular glands (green box), the mucosa with dysplastic changes had the disordered arrangement of glands and large hyperchromatic nuclei (red box), Scale bars = 2.5 mm. The fields in the green or red boxes were magnified and shown on the upper panel, Scale bars = 250 μm. **(E)** IHC was performed to examine the expression of CEACAM6 in the dysplastic gastric mucosa got from ESD operation, the tissues expressed CEACAM6 were stained in brown, Scale bars = 2.5 mm, and the fields in the red or green box were magnified and shown on the upper panel, Scale bars = 250 μm. **(F)** The histograms showed the quantification of IHC staining, the data are expressed as the mean (± SEM) of staining H-Score (n = 15, mild dysplasia=5, moderate dysplasia=5, severe dysplasia=5). ***P<0.001. All immunohistochemical sections were scanned by 3D HISTECH (pannoramic MIDI) and 10 randomly selected fields were checked under pannoramic viewer, the percentage of positive cells was analyzed by the densito quant software and the staining H-Score was calculated.

According to the data provided above, CEACAM6 mRNAs were over expressed in both the Dys and Cancer groups. To detect the CEACAM6 protein in the GC, we sought to detect the expression patterns of CEACAM6 in GC tissue chips using IHC staining. The results showed that the positive rate of CEACAM6 was 66.29% (59/89) and 4.49% (4/89) in the tumor tissues and the adjacent tumor tissues, respectively, and the staining H-Score in tumor was 39.56 ± 6.01 (**Figures 2A, B**). From the clinical data shown in **Table 2**, patients with TNM classifications of stage T3-4 have higher CEACAM6 expression than those with stages T1-2 (P=0.026), and the patients with pathological classifications of grades 3-4 have higher CEACAM6 expression than those with grade 2 (P=0.007). Furthermore, the data showed that distant metastasis and N classification were closely related with the prognosis of patients and were independent risk factors for GC prognosis.

**Table 2:** The relationship between expression of CEACAM6 and clinicopathological features of GC.

Clinicopath-ological features	Group	N	Expression of CEACAM6	*p*
Total		89	1.618±2.147	0.000
Age(years)	≥50	82	1.683±2.217	0.331
	<50	7	0.857±0.690	
Sex	male	62	1.645±2.057	0.866
	female	27	1.556±2.379	
metastasis	no	87	1.655±2.157	/
	yes	2	0	
Depth of invasion	Serosa inner	63	1.611±2.197	0.963
	Serosa outer	26	1.635±2.062	
T staging	T1-2	14	0.929±0.896	**0.026**
	T3-4	75	1.746±2.287	
N staging	N0	21	1.643±2.180	0.498
	N1-4	68	1.610±2.152	
Pathological grading	II	17	0.853±0.843	**0.007**
	III-IV	72	1.799±2.319	
AJCC clinical staging	1-2	33	1.500±2.118	0.693
	3-4	56	1.687±2.180	
Tumor diameter(cm)	≤5	45	1.500±1.787	0.603
	>5	44	1.739±2.477	

Bold values, P < 0.05.

During the end point of 60 months of follow-up, a total of 51 patients died whereas the other 38 remained alive. The survival analysis showed that the patients with higher positive CEACAM6 expression have shorter survival time than those with lower CEACAM6 expression (**Figure 2C**). To investigate the CEACAM6 expression in dysplastic gastric mucosa, tissues of patients with mild, moderate or severe dysplasia obtained from the ESD procedures were stained with anti-CEACAM6 mAb using IHC staining. The results show that compared to the adjacent gland, CEACAM6 was highly expressed in the gland with dysplastic changes (**Figures 2D–F**). *** P <0.001.

### CEACAM6 Promoted the Invasive, Metastatic, and Proliferative Ability of GC Cells

**Figure 3:**
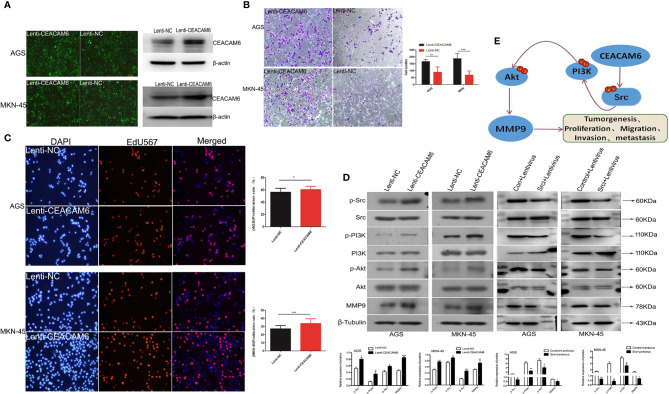
CEACAM6 promoted the GC cells invasion, proliferation and metastasis. **(A)** AGS and MKN-45 cells were infected with CEACAM6 lentivirus (Lenti-CEACAM6) or negative control lentivirus (Lenti-NC), the cells infected with lentivirus were showed in green color when checked with fluorescent microscopy, and the infection efficiency was also checked with western-blot. **(B)** After 48 h of infection, 2.5x10^4^ cells were trypsinized and seeded in 24−well plates with matrigel−coated membranes for the invasion assays. The upper panel represented the AGS cells and the down panel represented the MKN-45 cells. The number of cells in 10 randomly selected fields was counted at 48 h after incubation. Data represents the mean value of three independent experiments. Bars represent the mean (± SEM) number of invaded cells per field. **P < 0.01, ***P < 0.001. **(C)** 3x10^4^ cells/ml were seeded in 96−well plates and grown for 24 h and then infected with Lenti-CEACAM6 and Lenti-NC for 48 h. Cells were stained with EdU−Apollo 567 and DAPI; DAPI blue fluorescence represents all live cells and the EdU−Apollo567 red fluorescence represents the proliferating cells. The number of cells in 10 randomly selected fields were counted. The proliferation ratio was calculated as number of proliferating cells/total number of cells x 100%. Mean (± SEM) values of three independent experiments are presented.*p<0.05, ***p<0.001. **(D)** Western-blot assays were performed to detect the expression of PI3K, p−PI3K Akt, p−Akt, Src, p-Src and MMP9. β−Tubulin was used as the internal control for total proteins, while total protein was used as the internal control for the phosphorylated protein. Mean (± SEM) values of three independent experiments are presented, *p<0.05, **P<0.01, ***p<0.001. Control: Src inhibitor control, Lentivirus: CEACAM6 over expression lentivirus (Lenti-CEACAM6), Srci:Src inhibitor. **(E)** The possible pattern graph of CEACAM6 to Src/PI3K/Akt signaling pathway.

In order to study the roles of CEACAM6 in GC cells, the AGS and MKN-45 cells were infected with Lenti-CEACAM6 or Lenti-NC. The infection efficiency was checked using fluorescence microscopy and western-blot (**Figure 3A**), and the cell invasion and proliferation were detected 48 h after infection. The transwell assay results showed that CEACAM6 over expression promoted GC cell invasion (**Figure 3B**). Moreover, the EdU assay showed that CEACAM6 can promote the proliferation of GC cells (**Figure 3C**). *P<0.05, **P<0.01, *** P <0.001.

Studies found Src/PI3K/Akt signaling pathway activation is responsible for the GC tumorigenesis (15, 16), furthermore, the western-blot was applied to study the roles of CEACAM6 in proteins of Src/PI3K signaling pathway, with the results showing that CEACEM6 over expression can up regulate the expression of p-Src, p-PI3K, p-Akt and MMP9 proteins in the AGS and MKN-45 cells, however, when the Src was inhibited, the down regulation of p-PI3K, p-Akt and MMP9 cannot be recovered by CEACAM6 (**Figure 3D**). It was speculated that CEACAM6 can accelerate the GC tumorigenesis and progression by Src/PI3K/Akt signaling pathway (**Figure 3E**).

### Over Expression of CEACAM6 Promoted GC Cells Derived Tumor Growth

**Figure 4:**
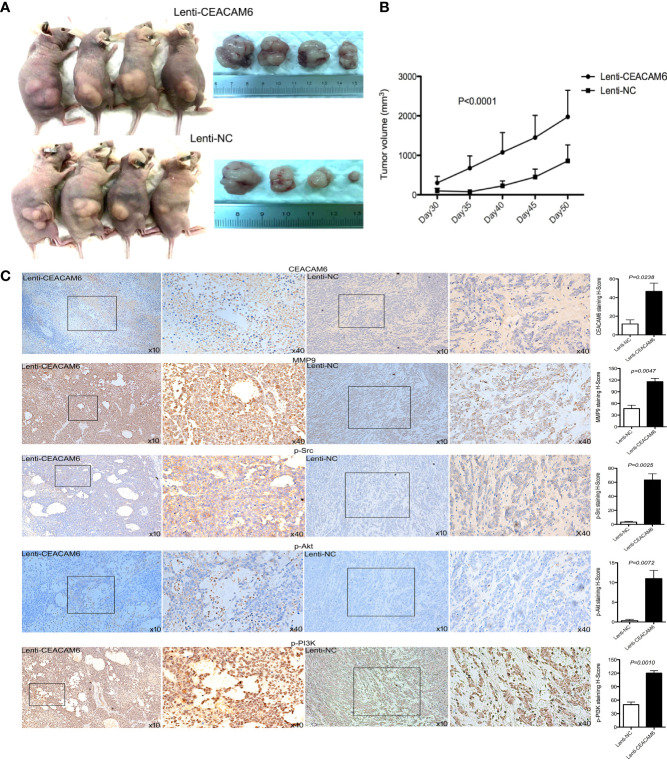
The effect of CEACAM6 on cell derived tumorigenesis. **(A)** Approximately 4 × 10^7^ MKN-45 cells infected with Lenti-CEACAM6 or Lenti-NC were inoculated subcutaneously into the mice, the MKN-45 cell derived subcutaneous tumors in a xenograft mouse model was constructed. **(B)** 30 days after the inoculation, the width and length of tumor were measured and the volume was calculated *in vivo* every 5 days till day 50. **(C)** At the day 50, the mice were euthanasia, the subcutaneous tumors were harvested for IHC staining of p−Akt, p−PI3K p−Src, and MMP9. The images in the left panel were magnified 100 folds (x10), and images in the right panel are a magnification of the indicated black boxes, at 400 folds magnification (x40), the positive staining was showed in the brown. All IHC sections were scanned by 3D HISTECH (pannoramic MIDI) and 10 randomly selected fields were checked under pannoramic viewer, the percentage of positive cells was analyzed by the densito quant software and the staining H-Score was calculated and showed with histograms (right panel). The data are expressed as the mean (± SEM) of staining H-Score from three independent experiments.

To further investigate the role of CEACAM6 in GC cell tumorigenesis *in vivo*, the subcutaneous tumors from CEACAM6 over expressed MKN-45 cells in a xenograft mouse model were studied. We found that CEACAM6 (Lenti-CEACAM6) over expression led to a significant increase in the tumor volume in a time-dependent manner, as compared to the Lenti-NC group (**Figures 4A, B**). From the data obtained above, it was found that CEACAM6 can increase the expression of p-Src, p-PI3K, p-Akt, and MMP9 proteins. Then, we checked the expression of these proteins in the subcutaneous tumor tissues and found that high CEACAM6 expression can also promote the expression of p-Src, p-PI3K, p-Akt, and MMP9 proteins *in vivo* (**Figure 4C**). The results further confirmed that the activation of the Src/Akt/PI3K/MMP9 signaling pathway may be a mechanism by which CEACAM6 promotes GC cell metastasis *in vivo*

### The Anti-CEACAM6 Probe Has High Affinity to CEACAM6

**Figure 5:**
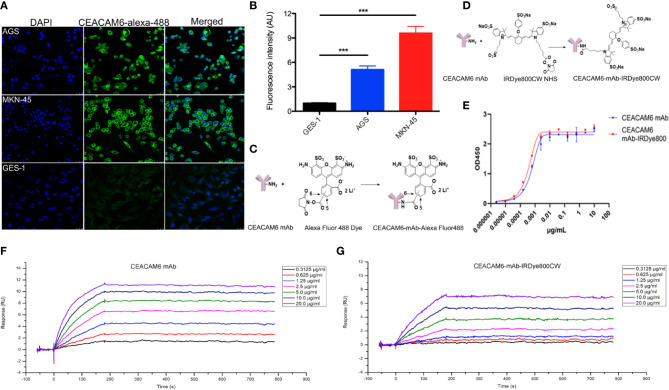
The affinity of CEACAM6-mAb-Alexa Fluo488 probe to GC cells and the CEAACM6-mAb-IRDye800CW probe tracked the tumor in mice model. **(A)** The AGS, MKN-45 and GES-1 cells were cultured and incubated with CEACAM6-mAb-Alexa Fluo488 probe (1:20, green signal), the nucleus were stained with DAPI (blue signal), 10 randomly selected fields were checked under confocal microscope. **(B)** Quantification of the mean fluorescence intensity in the cells by Image-Pro Plus software (fold change from GES-1), and the bar graph was drawn, data are expressed as the mean (± SEM) of values from three independent experiments. ***p < 0.001. **(C)** The chemicalmolecular modeldiagram of CEACAM6-mAb-Alexa Fluor488 was showed. **(D)** The chemicalmolecular model diagram of CEACAM6-mAb-IRDye800 was showed. **(E)** The activity of CEAACM6-mAb-IRDye800CW was evaluated by ELISA method, the optical intensity was measured by spectrophotometer (OD450nm). The Y-axis respresented the OD value, the X-axis represented the different concentration of antibody, the fitting curve was generated based on the OD value and the IC50 was calculated according to the fitting curve by GraphPad Prism 8.0.2. **(F, G)** The surface plasmon resonance (SPR) analysis was used to test affinity of CEACAM6 mAb and CEACAM6 mAb-IRDye800CW to CEACAM6 respectively, Y-axis respresented the fluorescence signal value, the X-axis represented the times, the different diluted concentration of antibody were showed in the different colors.

In order to check the affinity of the fluorescent-labeled anti-CEACAM6 probe with GC cells, the CEACAM6-mAb-Alexa Fluo488 probe was synthesized, the AGS, MKN-45, and GES-1 cells were cultured in confocal specific dishes, and the cells were incubated with the fluorescent-labeled anti-CEACAM6 probe (CEACAM6- mAb-Alexa Fluo488). After incubation, the cells were observed under confocal microscopy. It was found that the probe was assembled on the cell membrane, as expressed by the CEACAM6 (green fluorescence) (**Figure 5A**). Moreover, the fluorescence intensity was highest in the MKN-45 cells (**Figure 5B**). ***P<0.001. The chemical molecular structure model diagram of CEACAM6-mAb-Alexa Fluo488 was showed in **Figure 5C**. It was suggested that the probe had the strongest affinity to MKN-45 cells.

The near infrared-labeled anti-CEACAM6 probe (CEACAM6 -mAb-IRDye800CW) was synthesized to study the tracking role of the anti-CEACAM6 probe in GC *in vivo*, the chemical molecular structure model diagram of CEACAM6 -mAb-IRDye800CW was showed in **Figure 5D**. The activity of the probe was evaluated using the ELISA method. The results showed that the CEACAM6 antibody labeled with IRDye800CW has the similar IC50 value (0.3501 ng/μl) with that of the CEACAM6 antibody without labeling (the IC50 value was 0.5705 ng/μl). Therefore, it was indicated that the activity of the CEACAM6-mAb-IRDye800CW was high and was not influenced by conjugates (**Figure 5E**).

In order to further evaluate the affinity of CEACAM6-mAb-IRDye800CW to CEACAM6, the surface plasmon resonance (SPR) analysis was performed. For CEACAM6-mAb, the association (Ka) rate constant was 1.422×10^5^ M^-1^s^-1^, the dissociation (Kd) rate constant was 2.2876×10^-5^s^-1^, and the average dissociation constant (KD) was 2.022×10^-10^ M (**Figure 5F**). For CEACAM6-mAb-IRDye800CW, the Ka rate constant was 4.996×10^4^ M^-1^s^-1^, the Kd rate constant was 2.989×10^-5^ s^-1^, and the KD was 5.983×10^-10^ M (**Figure 5G**), from the data above, it was showed the CEACAM6-mAb-IRDye800CW had the similar Kd rate constant to CEACAM6 mAb, the Ka rate constant of CEACAM6-mAb-IRDye800CW was slightly lower than CEACAM6 mAb, then it was found the affinity of labeled antibody was slightly lower than unlabeled antibody accord to the KD value.

### The CEACAM6 -mAb-IRDye800CW Can Track the Tumor as Well as Metastases *In Vivo*

**Figure 6:**
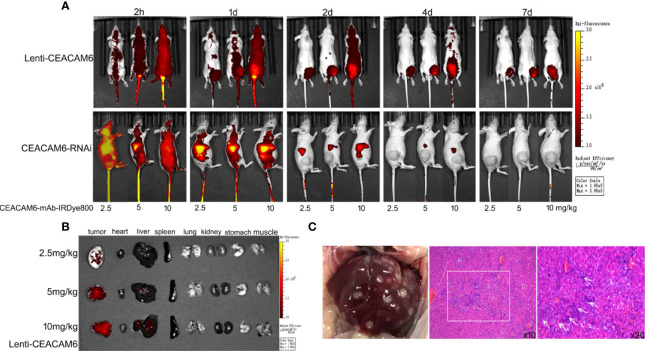
**(A)** The MKN−45 cells infected with Lenti-CEACAM6 or CEACAM6 RNAi were used to derive the subcutaneous tumor mice model, for the *in vivo* imaging study, the CEAACM6-mAb-IRDye800CW probe with the concentration of 2.5, 5 and 10 mg/kg was injected in the caudal vein of the mice respectively, the fluorescence intensity were observed at 2 h until 7 days, and the tissues which were expressed the CEACAM6 highly were labeled by the probe and showed the red signals. **(B)** After imaging, the mice were euthanasia the tumor, heart, liver, spleen, lung, kidney, stomach and muscle were removed and imaged, the tissues which were expressed the CEACAM6 highly were labeled by the probe and showed the red signals. **(C)** The surface change of the liver got above, the HE staining was applied to study the histology of liver, the metastases was showed in the white box, and the cancer cells were indicated with white arrows.

The anti-CEACAM6 demonstrated strongest affinity to the MKN-45 cells, hence the MKN-45 cells infected with Lenti-CEACAM6 or CEACAM6-RNAi were used for the xenograft mouse GC model construction and *in vivo* study. The CEACAM6-mAb-IRDye800CW probe was injected into the mouse model through the caudal vein and the whole-body imaging was acquired using animal computed tomography. When the CEACAM6-mAb-IRDye800CW with different dose was injected in the caudal vein of the mice, the signal intensity was observed from 2 h up to 7 days. In the Lenti-CEACAM6 group, the signal intensity was enhanced with the dose increasing but was attenuated with passing time. Therefore, the fluorescence signals were strongest at 2 h with the dose of 10 mg/kg. In the CEACAM6 RNAi group, the signal intensity showed the similar change pattern from 2 h to day 7, but it was more weak when compared with Lenti-CEACAM6 group, the signal was very weak at the day 4 and there was almost non signal was detected at day 7 (**Figure 6A**). The tumor, heart, liver, spleen, lung, kidney, stomach, and muscles from Lenti-CEACAM6 were removed from the mice at day 7. The highest signal intensity was observed in the tumor with the dose of 10 mg/kg CEACAM6-mAb-IRDye800CW probe injection. The signal was also observed in the liver (**Figure 6B**), it was speculated that the tumor has metastasized to the liver. The result was supported by the HE staining that the surface of the liver became spotty, the arrangement of hepatic lobules was disordered, there were metastases in the tissue (white box) and a large number of cancer cells infiltrated (white arrows) (**Figure 6C**).

### The CEACAM6-mAb-IRDye800CW Probe Can Label Dysplasia in Gastric Mucosa Obtained From ESD Operation

**Figure 7:**
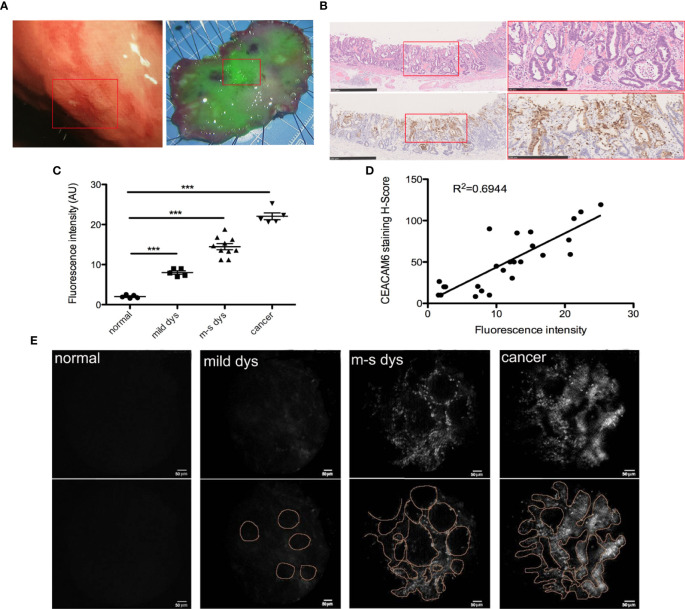
CEAACM6-mAbIRDye800CW probe labeled the gastric mucosa with dysplasia. **(A)** The white light endoscopy of the lesion in upper gastric body (left panel), the ESD was carried out and the specimen was got, the specimen was fixed on the board with pin on the edges, and flushed with running tap water to remove the mucus on the surface, then the CEAACM6-mAb-IRDye800CW probe was sprayed on the surface of the specimen evenly and incubated, then the detector of the wide field fluorescent endoscopy was used to check the fluorescent intensity, the mucosa binding the probe showed the green fluorescent signals, the intensity of the signals indicated how much the probe be binded. The depressed gastric mucosa at the center of the specimen showed the strongest green signals and was signed in the white box. **(B)** After fluorescent probe study, the specimen was fixed with formalin and the HE staining were investigated (upper panel), the depression was signed with red box (left panel) and was amplified (right panel), the glands were irregular and the nucleus were large and hyperchromatic. IHC staining of CEACAM6 in the specimen of ESD operation (down panel). The depressed gastric mucosa was showed in the red box (left panel) and was amplified (right panle), the positive of CEACAM6 expression was showed in the brown. Scale bars in left panel = 500 μm, Scale bars in right panel = 250 μm. **(C)** The mean fluorescence intensity in the gastric mucosa with different lesions was quantified by Image-Pro Plus software (fold change from normal), Data are expressed as the means ± SD, the normal mucosa=5, mucosa with mild dysplasia=5, mucosa with moderate dysplasia=5, mucosa with severe dysplasia =5 and mucosa with cancer=5, m-s dys: moderate or severe dys. ***P < 0.001. **(D)** The correlation between fluorescence intensity and CEACAM6 IHC staining index was studied by Pearson correlation coefficients. **(E)** The micro structure of gastric mucosa surface was observed by the fluorescent micro endoscopy detector. The surface micro structure was showed in upper panel and the pattern diagrams were drawn (low panel).

From the data described above, it was shown that the anti-CEACAM6 probe has high affinity with GC cells and can track the tumor as well as the metastases *in vivo* in the mice model. Therefore, we performed the analysis of gastric specimens with dysplasia after ESD operations. The hand-held fluorescent detector was used to check the fluorescent signals. The CEACAM6-mAb-IRDye800CW probe could label the neoplastic lesion exactly (**Figure 7A**), and the mucosa with the strongest fluorescence intensity had the highest CEACAM6 expression when compared with adjacent mucosa (**Figure 7B**). The fluorescence intensity was increasing with the pathological changes progression of the mucosa, in another words, the mucosa with the cancer had the strongest fluorescence intensity (**Figure 7C**). Furthermore, it was showed that the fluorescence intensity closely related with the CEACAM6 staining H-Score (R^2 ^= 0.6944) (**Figure 7D**). Meanwhile, the surface micro structure of the gastric mucosa was detected using the hand-held detector of the fluorescent microendoscopy. It was investigated the normal mucosa had no signal, the mucosa with mild dysplasia had weak signals that looked similar to regular circles, the mucosa with moderate to severe dysplasia had stronger signals than that with mild dysplasia, with the signals having an appearance similar to irregular circles. The mucosa with the adenocarcinoma had the strongest signal, and the signals characterized as messy (**Figure 7E**).

## Discussion

GC is the one of the most common tumors in digestive system, and is the second and third in incidence and mortality worldwide, respectively (2, 3, 17, 18). The EGC can be cured by endoscopic resection (ESD) and has the good prognosis, but the Non-early operable GC is treated by surgery and has the low 5-year survival (19). Thus, the early detection of lesions is very important. Although the invention of new types of endoscopies provided useful tools for tumor diagnosis, the accurate tool for EGC diagnosis is still lacking; therefore, there is a very urgent need to find an endoscopic method for EGC diagnosis that is easy and independent from the operator.

Antibodies conjugated with fluorescent dyes can image tumors by targeted optical imaging (20), the fluorescent probe is a new method that can visualize tumors (21). It was found the novel zinc ion fluorescent probe DPP-C2 showed potential application for the early detection of prostate cancer in tumor-bearing nude mice (22). Studies showed Cathepsin B (CB)-activated polymeric probe, P-(GGFLGK-IR783), can detect the colorectal cancer as well as polyps in mice model (23). Then, the probe OTL38 was targeted to folic acid, Bevacizumab-IRDye800CW was targeted to vascular endothelial growth factor, and BLZ-100 was targeted to annexin were used during endoscopic-guided tumor screening and intraoperative navigation; moreover, some of these probes have been under successive clinical trials (24–27). Therefore, special molecular fluorescent probe targeting could be the trend for early tumor diagnosis.

Biomarkers are very important in molecular imaging diagnosis. CEACAM6 is one of the members of the CAMCAMs family, is mainly expressed on the cell membrane, and is over expressed in various solid tumors (28–30). It was also reported that CEACAM6 expression was higher in pancreatic cancer with low differentiation than in medium differentiation, similarly, it was found that CEACAM6 expression was higher in tumor tissues than in adjacent normal tissues in colon cancer (31). An increasing number of studies have found that CEACAM6 plays an important role in GC progression and that it is a prognostic biomarker and potential therapeutic target for GC (32, 33).

Src is the prototypal member of Src Family tyrosine Kinases (SFKs), as the oncogene, it plays an important role in solid cancers, it promotes tumor growth and formation of distant metastasis (34, 35). Src is found to be one of the key pathway to regulate the GC carcinogenesis  (36), it can accelerate the GC progression by MMP9 (37). In the current study, according to TCGA data and our tissues sequencing data, CEACAM6 was found over expressed in the GC tissues compared to the adjacent normal tissues.

Meanwhile, CEACAM6 showed over expressed in dysplastic gastric mucosa tissues. Then it was demonstrated that CEACAM6 can promote the proliferation and invasion ability of GC cells through *in vitro* as well as *in vivo* studies. Over expression of CEACAM6 was found to promote the p-Src, p-PI3K, p-Akt and MMP9 proteins expression, when the Src signaling pathway was inhibitor, CEACAM6 cannot recover the down regulation of these proteins. It was speculated that CEACAM6 accelerate GC carcinogenesis by Src/PI3K/Akt/MMP9 signaling pathway. These are consistent with the results of previous studies showing that up regulated CEACAM6 can accelerate the GC cells invasion through the TGF-β, AkT, FAK, or Src signaling pathways (12, 38), CEACAM6 can active Akt signaling pathway in a Src-dependent manner in the pancreatic cells (39). Studies showedCEACAM6 was over expressed in the tumor or dysplasia tissues compared to adjacent normal tissues, with the area under curve being 0.83 when it was used to diagnose stage T1 GC, this study used the fluorescent jointed anti-CEACAM6 probe (CEACAM6-mAb-Alexa Fluor488) to label the gastric tumor obtained from PDTX, with the results showing that the probe can closely integrate with the GC tissues and very accurately label the tumor (13). CEACAM6 provided a potential method for GC diagnosis by endoscopy, however, there were no further studies reporting its use in precancerous diagnosis.

Years ago, a study has investigated the vital-dye enhanced fluorescence imaging of metaplasia, neoplasia and inflammation of GI mucosa, it was demonstrated that vital-dye enhanced fluorescence can identify the different lesions of GI and provide real-time, *in vivo* diagnoses (40), but the specific targeting probe has not been used. In our study, the CEACAM6- mAb-Alexa Fluor488 probe was synthesized to test the affinity of the anti-CEACAM6 probe with the GC cells. Because the near-infrared fluorescence (NIRF) imaging agents are found to be promising tools for noninvasive GC imaging (41, 42), on the other hand, the near infrared CEACAM6 –mAb-IRDye800CW probe was synthesized for the *in vivo* tracing study. The results showed that the anti-CEACAM6 probe can closely bind to the GC cells and can track the tumor in the MKN-45 cells derived GC mice model. Therefore, in order to demonstrate the diagnostic role of the probe in dysplastic mucosa, fresh specimens obtained from ESD operations were used for the first time. From the wide field of fluorescent endoscopy, it was shown that the CEACAM6-mAb-IRDye800CW probe can label the dysplastic mucosa precisely, the fluorescence intensity was increased gradually in the gastric mucosa from normal to cancerous, and the fluorescence intensity closely related to the CEACAM6 protein expression. Furthermore, it was demonstrated that the mucosa with different degrees of dysplasia or cancer has different surface structures by fluorescent micro endoscopy. It was speculated that the CEACAM6-mAb-IRDye800CW probe can diagnosis the precancerous lesions especially can distinguish the mucosa with CAG+IM to Dys.

Nevertheless, our work still has some limitations. There were only 15 dysplastic specimens used for this study. In the future, more samples should be used, and the CEACAM6 expression in different degrees of dysplastic mucosa should also be studied. Moreover, the results that we have obtained were only from a pilot study, further studies are needed to verify the diagnostic role of the anti-CEACAM6 probe in precancerous mucosa, and the ROC curve analysis needs be done to evaluate the sensitivity and specificity of anti-CEACAM6 probe.

In conclusion, CECAM6 is a potential biomarker for EGC, and the anti-CEACAM6 probe may provide a new endoscopic diagnostic method for EGC in the future.

## Data Availability Statement

The datasets presented in this study can be found in online repositories. The names of the repository/repositories and accession number(s) can be found in the article/supplementary material.

## Ethics Statement 

The studies involving human participants were reviewed and approved by Institutional Ethics Committee of Nanjing Medical University. The patients/participants provided their written informed consent to participate in this study. The animal study was reviewed and approved by Institutional Ethics Committee of Nanjing Medical University.

## Author Contributions

QZ and FA designed experiments, wrote and edited the manuscript. CZ, GZ, KC and LZ conducted the experiments. ZH designed the probes. YW designed the fluorescent endoscopy. ZZ analyzed the pathologies. FA analyzed the data and made figures. All authors contributed to the article and approved the submitted version.

## Funding

This study was supported in part by grants from the Major Project in Wuxi (no. Z201903 to FA), the Top Talent Project in Wuxi (no. BJ2020003 to FA), the Wuxi Science and Technology Bureau Project (no. N20201004 to QZ), the National Natural Science Foundation of China (no. 81773227 to QZ), and the high-end medical team of the Wuxi “Taihu Talent Project” (to QZ).

## Conflict of Interest

The authors declare that the research was conducted in the absence of any commercial or financial relationships that could be construed as a potential conflict of interest.
